# A Rare Co-occurrence of Monkeypox Encephalitis and Neurosyphilis

**DOI:** 10.7759/cureus.35945

**Published:** 2023-03-09

**Authors:** Rohit Sharma, Tristan Nguyen-Luu, Pragya Dhaubhadel, Amit Sharma, Roopa Naik

**Affiliations:** 1 Medicine, Geisinger Health System, Wilkes-Barre, USA; 2 Clinical Research, Dresden International University, Dresden, DEU; 3 Internal Medicine, Geisinger Health System, Wilkes-Barre, USA; 4 Infectious Disease, Geisinger Health System, Scranton, USA

**Keywords:** monkeypox-associated cns disease, chatgpt improved case report, chatgpt, monkeypox coinfection, monkeypox encephalitis, neurosyphillis, monkeypox virus

## Abstract

Monkeypox (MPOX according to the Centers for Disease Control and Prevention) has been a disease of interest in populations with high-risk sexual behavior. As sporadic outbreaks of MPOX have led to a worldwide spread, it has been declared a public health emergency by the World Health Organization. Here, we describe the case of a 44-year-old male with high-risk sexual behavior who presented with typical rashes of MPOX and altered mental status. MPOX polymerase chain reaction from the skin lesion and cerebrospinal fluid-Venereal Disease Research Laboratory tests were positive, raising the possibility of concomitant infection with neurosyphilis. The patient was treated with tecovirimat and aqueous penicillin G resulting in an improvement in the patient’s clinical condition. Our case describes that MPOX has the potential to cause central nervous system manifestations through possibly a direct viral invasion or an immune-meditated insult.

## Introduction

Monkeypox (MPOX) is a rare viral infection that is caused by the MPOX virus, belonging to the Orthopoxvirus genus. It was first identified in 1958 in laboratory monkeys, with subsequent infections in humans only reported in the 1970s. MPOX is endemic to Central and West African countries. Although it is predominantly seen in animals, sporadic outbreaks in humans have been reported.

The clinical presentation of MPOX is similar to smallpox, albeit less severe. The disease is generally self-limiting and is characterized by fever, headaches, myalgias, and characteristic pox-like skin lesions. A majority of patients recover within a few weeks, however, complications such as pneumonia, sepsis, and neurological disease can occur. The mortality rate associated with MPOX is approximately 1-10%.

Neurological complications of MPOX are relatively rare but can be serious and potentially fatal [1]. The most common neurological complication is encephalitis. Other neurological complications that have been reported include transverse myelitis and optic neuritis [2-4].

One case of MPOX and neurosyphilis has been reported recently but the cerebrospinal fluid-Venereal Disease Research Laboratory (CSF-VDRL) was negative and only rapid plasma reagin (RPR) was positive [5]. Although the mechanism of neurological disease in MPOX is not well understood, it is believed to be related to direct viral invasion of the nervous system, acute perivenular demyelination, as well as an immune response to the virus [6]. The risk of neurological disease appears to be higher in individuals with certain risk factors, such as immunocompromised individuals, pregnancy, and individuals with a history of skin lesions or other poxvirus infections [6].

## Case presentation

**Figure 1:**
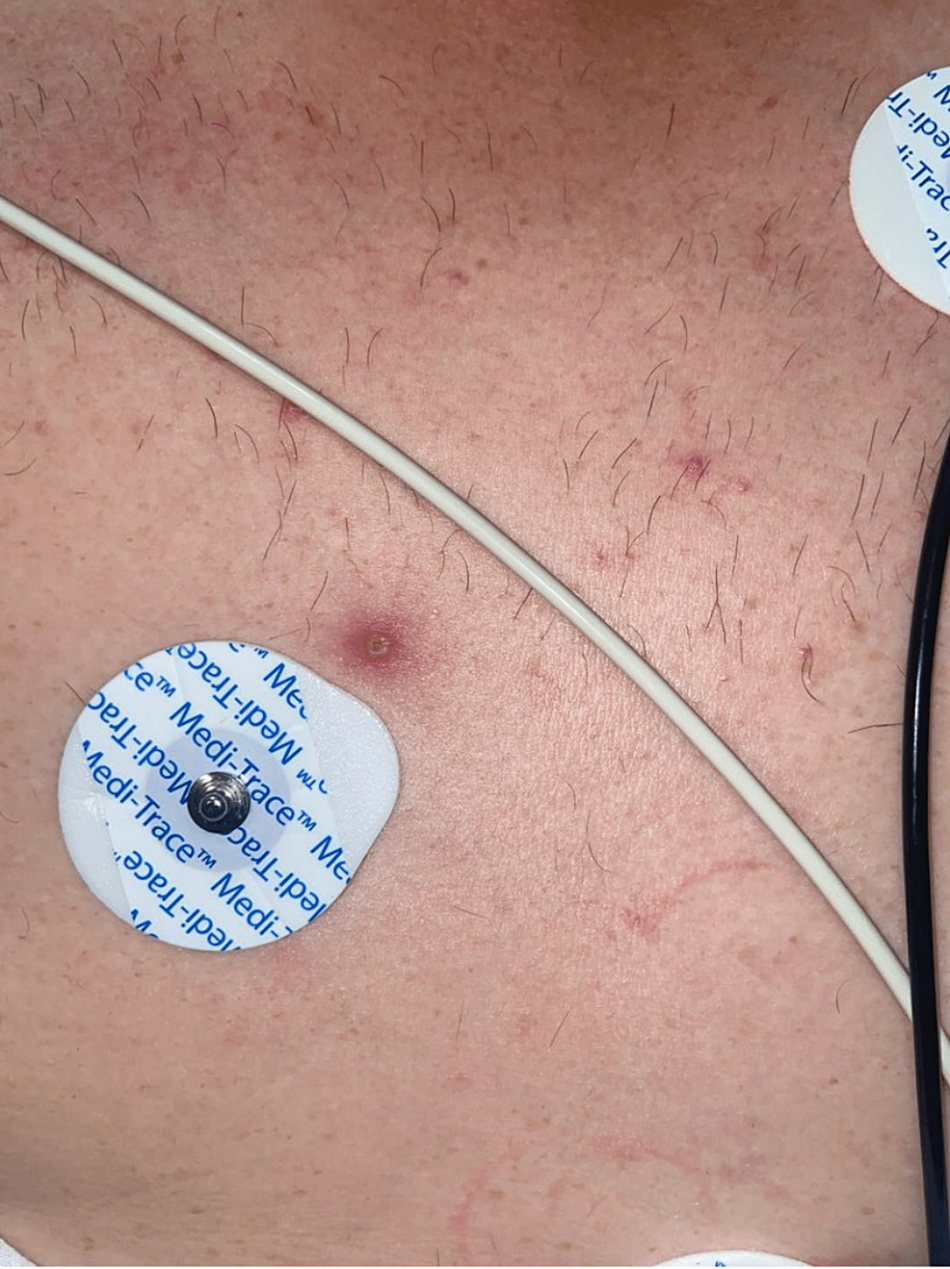
Characteristic lesion of monkeypox on the chest.

**Figure 2:**
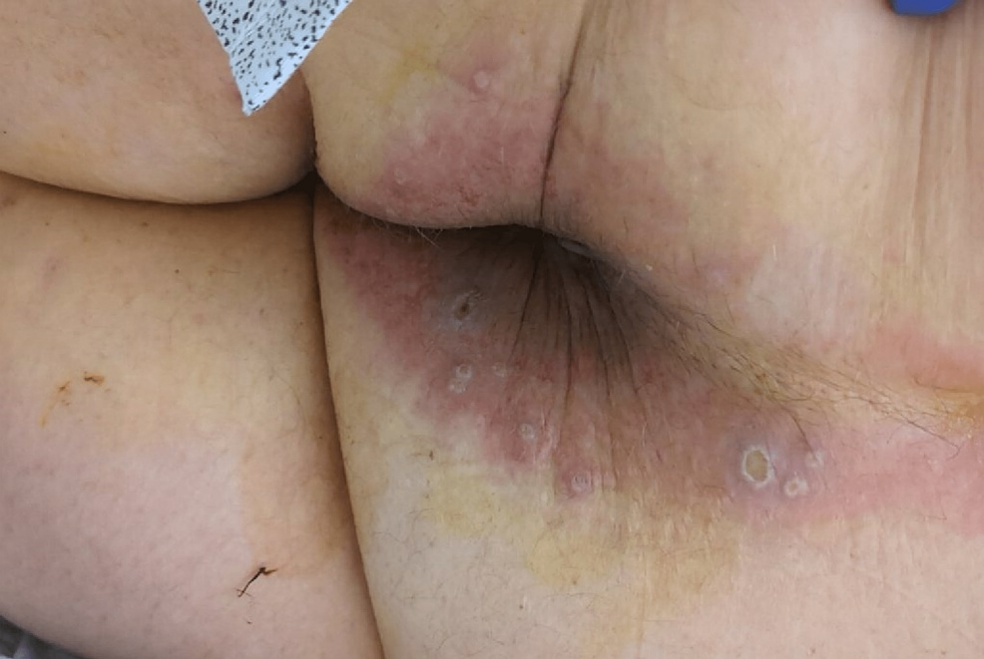
Multiple ulcerated monkeypox lesions around the anal region.

A 44-year-old male with a past medical history of high-risk sexual behavior (men having sex with men, MSM) on pre-exposure prophylaxis for HIV, a history of neurosyphilis, and genital herpes on valacyclovir presented to the hospital with altered mental status. Neurosyphilis was previously diagnosed due to the presence of ocular complications leading to high intraocular pressure. He was treated with aqueous penicillin G. The patient was found to be awake, staring, and non-responsive, and was brought in by friends to the emergency department. Four days prior to the admission, the patient presented to the Infectious Diseases office with characteristic sharply raised, round, and pustular rashes of MPOX (Figures 1, 2). The lesion was swabbed which revealed a positive polymerase chain reaction (PCR) for MPOX DNA. On examination, vitals were within normal limits. Neurological examination revealed a Glasgow Coma Scale score of 12/15 (E4, V3, M5), not alert and oriented to time, place, and person; no neck rigidity; no meningeal signs; normal reflexes; and no obvious focal neurological deficits, although the examination was limited due to the patient being non-cooperative. Labs revealed mild leukocytosis 11.08 K/μL (4.00-10.80 K/μL), normal liver and kidney function, and urine toxicology negative for common drugs of abuse. Lumbar puncture was done with total nucleated cells being 32 leucocytes/μL (normal <5 leucocytes/μL), CSF protein 132 mg/dL (15-45 mg/dl), normal CSF glucose, negative CSF viral panel, negative CSF culture. The patient was also found to be CSF-VDRL positive (reactive 1:1, noted to be negative five months before the current admission), which could also potentially support a diagnosis of neurosyphilis. MRI of the brain with contrast revealed no acute intracranial abnormality. Electroencephalography revealed mild diffuse slowing and focal right temporoparietal slowing. The patient was treated with tecovirimat (600 mg every 12 hours for 14 days) and aqueous intravenous penicillin G (24 million units every four hours for 10 days). The patient had significant clinical improvement in clinical status along with improvement in mentation back to baseline. The patient was eventually discharged in stable condition.

## Discussion

This case underscores the importance of considering multiple potential etiologies for altered mental status in patients with a complex medical history, especially when there are multiple overlapping risk factors. In this case, the patient’s history of high-risk sexual behavior put him at an increased risk for multiple sexually transmitted infections, including HIV, neurosyphilis, and MPOX. It is recognized that lymphadenopathy, fatigue, and the characteristic rash are the symptoms with the greatest sensitivity (≥80%) for MPOX, whereas conjunctivitis and genital lesions are symptoms with high specificity [7]. The diagnosis of MPOX virus infection is relatively rare in the United States, and this case highlights the need for clinicians to maintain a high index of suspicion for rare infections, especially in individuals with a history of travel or high-risk MSM sexual behaviors. One case reported MPOX and secondary syphilis co-infection leading to delayed clearance of MPOX for up to 30 days possibly due to immune dysfunction [8]. MPOX-associated encephalitis was first reported in 2003 in a family outbreak with a six-year-old girl developing severe encephalitis [9]. To our knowledge, only one case has been reported with MPOX and suspected neurosyphilis as RPR was positive (1:64) while CSF-VDRL was negative [5]. Limited cases of MPOX have been known to have neurological involvement [10]. The central nervous system (CNS) complications in these cases are thought to occur either by an autoimmune process triggered by the infection or by an indirect inflammation in the CNS as a result of an exaggerated immune response by the body [10,11]. However, it is not possible to rule out the possibility of direct viral invasion in the CNS in our case due to a lack of data on the sensitivity and specificity of MPOX nucleic acid or serology tests available for use in the CSF. A positive CSF-VDRL test raises the possibility of neurosyphilis, which can cause a wide range of neurological symptoms, including altered mental status, headache, and focal neurological deficits.

## Conclusions

In populations at high risk, such as MSM, it is crucial to consider MPOX as a potential cause of neurological symptoms. Diagnosis may involve conducting MPOX PCR and CSF tests. Early initiation of antiviral therapy with tecovirimat is strongly recommended in the early stages of the disease to improve patient outcomes.

## Figures and Tables

**Figure 3:**
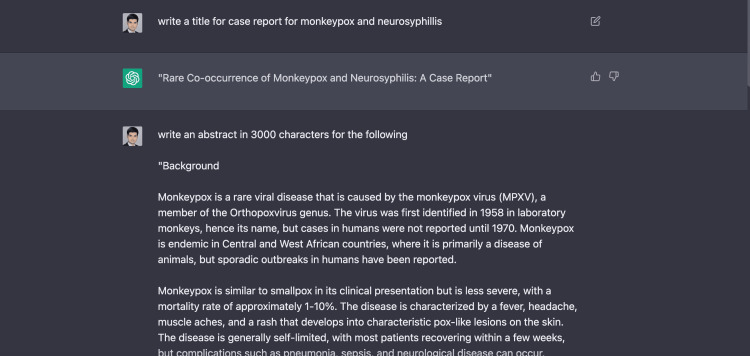
ChatGPT screenshot (i).

**Figure 4:**
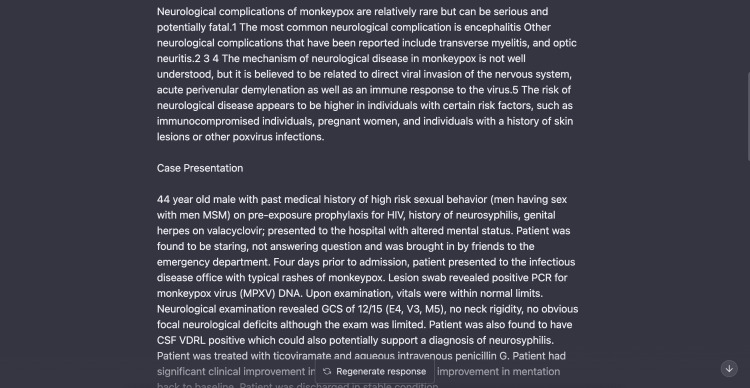
ChatGPT screenshot (ii).

**Figure 5:**
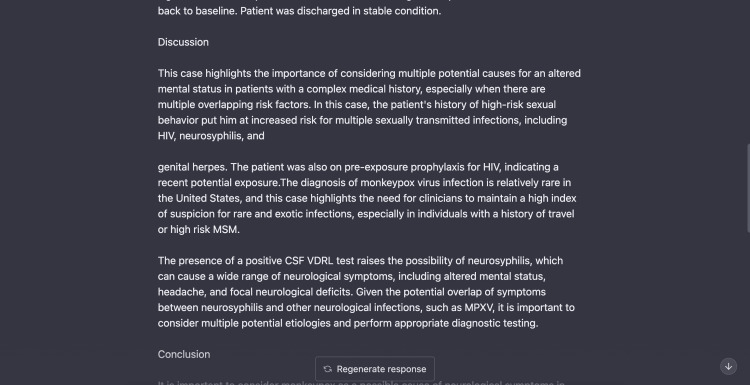
ChatGPT screenshot (iii).

**Figure 6:**
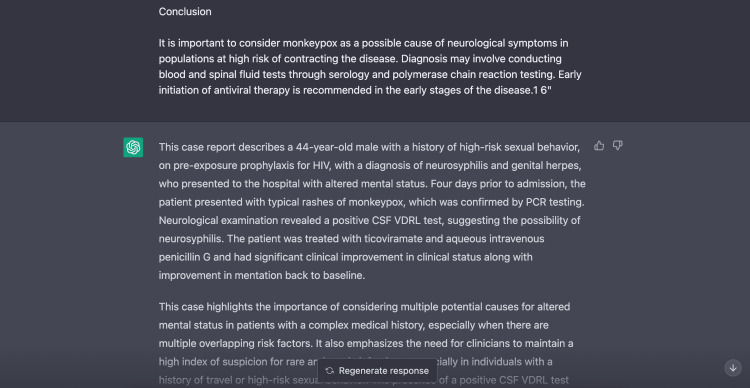
ChatGPT screenshot (iv).

**Figure 7:**
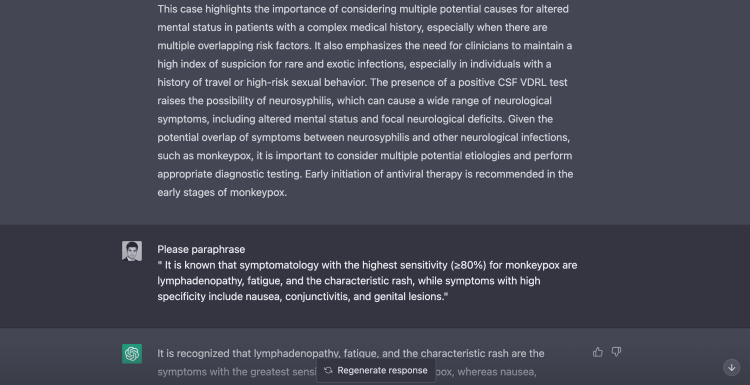
ChatGPT screenshot (v).

**Figure 8:**
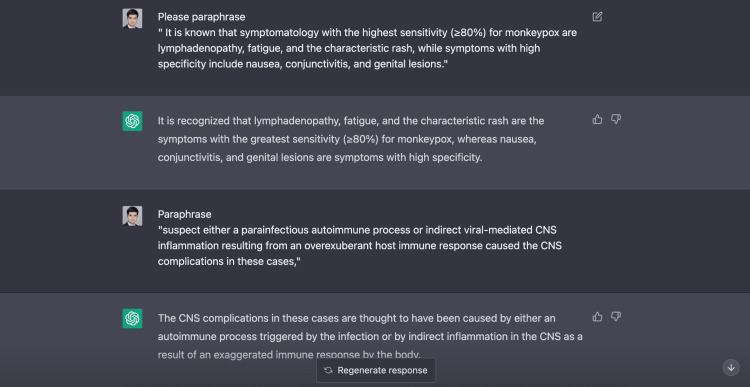
ChatGPT screenshot (vi).

**Figure 9:**
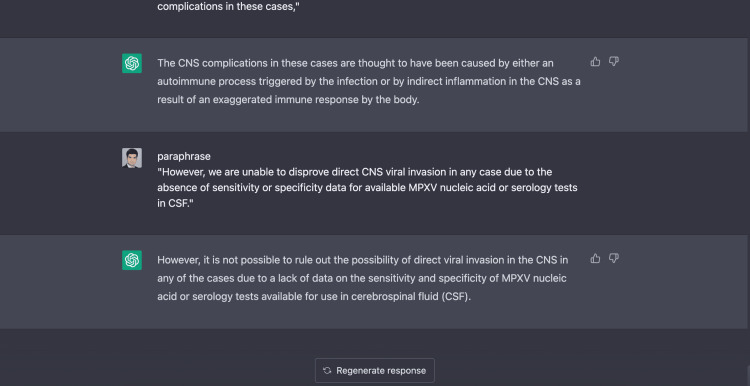
ChatGPT screenshot (vii).
